# Real-World Outcomes of Trastuzumab Deruxtecan in Patients With HER2+ Metastatic Breast Cancer: The DE-REAL Study

**DOI:** 10.1093/oncolo/oyad308

**Published:** 2023-11-23

**Authors:** Andrea Botticelli, Roberta Caputo, Simone Scagnoli, Simona Pisegna, Michelino De Laurentiis, Giuseppe Curigliano, Matteo Lambertini, Francesco Pantano, Antonella Palazzo, Ida Paris, Claudio Vernieri, Beatrice Tedesco, Marianna Giampaglia, Michela Palleschi, Zelmira Ballatore, Daniele Alesini, Giuliana D’Auria, Agnese Fabbri, Luigi Rossi, Annarita Verrazzo, Roberta Scafetta, Daniele Marinelli, Caterina Sposetti, Vittoria Barberi, Lidia Strigari, Paolo Marchetti, Daniele Santini, Alessandra Fabi

**Affiliations:** Department of Radiological, Oncological and Pathological Science, Sapienza University of Rome, Rome, Italy; Department of Breast and Thoracic Oncology, Division of Breast Medical Oncology, Istituto di Ricovero e Cura a Carattere Scientifico (IRCCS) Pascale, Naples, Italy; Department of Radiological, Oncological and Pathological Science, Sapienza University of Rome, Rome, Italy; Department of Experimental Medicine, Sapienza University of Rome, Rome, Italy; Department of Breast and Thoracic Oncology, Division of Breast Medical Oncology, Istituto di Ricovero e Cura a Carattere Scientifico (IRCCS) Pascale, Naples, Italy; Department of Oncology and Hematology, University of Milan, Milan, Italy; Division of New Drugs and Early Drug Development for Innovative Therapies, European Institute of Oncology, IRCCS, Milan, Italy; Department of Internal Medicine and Medical Specialties (DiMI), School of Medicine, University of Genova, Genova, Italy; Department of Medical Oncology, UOC Clinica di Oncologia Medica, IRCCS Ospedale Policlinico San Martino, Genova, Italy; Medical Oncology, Fondazione Policlinico Universitario Campus Bio-Medico, Rome, Italy; Depatment of Medical Oncology, Comprehensive Cancer Center, Fondazione Policlinico Universitario Agostino Gemelli, IRCCS, Rome, Italy; Department of Woman and Child Health, Fondazione Policlinico Universitario A. Gemelli IRCCS, Rome, Italy; Medical Oncology Department, Fondazione IRCCS Istituto Nazionale dei Tumori, Milan, Italy; IFOM ETS, the AIRC Institute of Molecular Oncology, Milan, Italy; Medical Oncology Unit, “S. Carlo” Hospital, Italy; Medical Oncology Unit, “S. Carlo” Hospital, Italy; IRCCS Istituto Romagnolo per lo Studio dei Tumori “Dino Amadori” IRST, Meldola, Italy; Clinical Oncology, Università Politecnica delle Marche, AOU Ospedali Riuniti, Ancona, Italy; UOSD Centro Oncologico S. Spirito e Nuovo Regina Margherita, Ospedale Santo Spirito in Sassia, Rome, Italy; Department of Medical Oncology, Sandro Pertini Hospital, RomeItaly; Department of Oncology and Hematology, Medical Oncology and Breast Unit, Central Hospital of Belcolle, Viterbo, Italy; Multispeciality Department of Oncology, ASL Latina, “Sapienza” University of Rome, Aprilia, Italy; Department of Breast and Thoracic Oncology, Division of Breast Medical Oncology, Istituto di Ricovero e Cura a Carattere Scientifico (IRCCS) Pascale, Naples, Italy; Medical Oncology, Fondazione Policlinico Universitario Campus Bio-Medico, Rome, Italy; Department of Radiological, Oncological and Pathological Science, Sapienza University of Rome, Rome, Italy; Medical Oncology Department, Fondazione IRCCS Istituto Nazionale dei Tumori, Milan, Italy; Sapienza Università di Roma - IRCCS Istituto Nazionale Tumori Regina Elena, RomeItaly; Department of Medical Physics, IRCCS Azienda Ospedaliero-Universitaria di Bologna, Bologna, Italy; Department of Oncology and Dermatological Oncology, Istituto Dermopatico dell’Immacolata IDI IRCCS, Rome, Italy; Department of Medico-Surgical Sciences and Biotechnology, Polo Pontino, Sapienza University of Rome, Rome, Italy; Medical Oncology A, AOU Policlinico Umberto I, Rome, Italy; Precision Medicine in Senology, Scientific Directorate - Department of Women and Child Health, Fondazione Policlinico Universitario “A. Gemelli” IRCCS, Rome, Italy

**Keywords:** HER2+, breast cancer, trastuzumab deruxtecan, DE-REAL study

## Abstract

**Background:**

Trastuzumab deruxtecan (T-DXd) demonstrated unprecedented efficacy in patients with pretreated HER2+ metastatic breast cancer (mBC). However, few data are available about its efficacy in routine clinical practice. In this multicenter retrospective study, we examined effectiveness and safety of T-DXd in a real-world population.

**Methods:**

Clinico-pathological information about patients with HER2+ mBC who received T-DXd were collected from 12 Italian hospitals. HER2 status was determined locally. Patients who received at least one administration of T-DXd, as any therapy line for advanced disease were included in the analysis. The primary endpoint was real-word PFS (rwPFS).

**Results:**

One hundred and forty-three patients were included. Median age was 66 (range: 37-90), and 4 men were included. Hormone receptor (HR) status was positive in 108 (75%) patients and negative in 35(25%). T-DXd was administered as first, second, third, or subsequent lines in 4 (3%), 16 (11%), 42 (29%), and 81 (57%) patients, respectively. Among 123 patients with measurable disease, the ORR was 68%, and the DCR was 93% (9 CRs, 74 PRs, and 30 SD). Nine (7%) patients had a primary resistance to T-DXd. With a median follow-up of 12 months, the median rwPFS was 16 months. RwPFS was 84%, 59%, and 39% at 6, 12, and 18 months, respectively. A favorable trend in rwPFS was reported in patients receiving T-DXd as I/II line versus further lines (17 vs. 15 months; *P* = .098). Any-grade toxicity was registered in 84 patients (59%). Most common adverse events (AEs) reported were nausea (33%), neutropenia (21%), and asthenia (21%). Liver toxicity and diarrhea were uncommon (5% and 1%). Severe toxicities was registered in 18% of patients, and the most frequent were neutropenia, nausea/vomiting, and ILD observed in 15, 2, and 3 patients. AEs led to dose reduction in 37 patients (26%). Dose reduction and AEs do not affect patients’ response and survival outcomes.

**Conclusions:**

Efficacy and safety of T-DXd were confirmed in an unselected real-world population of HER2+ mBC. These results are consistent with the results of known findings, and no new safety concerns were reported.

## Teaser

Trastuzumab deruxtecan (T-DXd) has shown efficacy in patients with pretreated HER2+ metastatic breast cancer; however, few data are available about its efficacy in routine clinical practice. In this multicenter retrospective study, effectiveness and safety of T-DXd was examined in a real-world population.

**Implications for Practice**

This study is a multicenter retrospective analysis involving a large cohort of patients with HER2+ advanced breast cancer. The authors were able to draw robust conclusions about the clinical benefits and safety profile of trastuzumab deruxtecan (T-Dxd) in real-world clinical practice. To date, few data are available about T-Dxd activity in a routine context. The DE-REAL study tried to fill this knowledge gap in order to optimize HER2+ metastatic breast cancer management in clinical practice.

## Background

Breast cancer (BC) is the most common malignancy among women, and the second cause of cancer-related mortality worldwide.^1^ Up to 20% of BCs harbor overexpression of Human Epidermal growth factor Receptor 2 (HER2) protein and/or amplification of the *HER2* gene. Of note, HER2-positive (HER2+) BC is an especially aggressive disease, and it is characterized by poor prognosis and more frequent relapses.^2,3^

In the last decades, the introduction of effective anti-HER2 agents has impressively improved the prognosis of patients with HER2+ BC, both in early and advanced disease settings.^4-6^ In the advanced disease setting, the CLEOPATRA trial demonstrated a consistent PFS and OS benefit by adding pertuzumab to trastuzumab and docetaxel in previously untreated patients with HER2+ BC.^5^ According to these data, the combination of pertuzumab, trastuzumab, and taxane-based chemotherapy has become the first-line standard of care for advanced HER2+ BC. For a decade, the standard second-line therapy has been represented by the antibody-drug conjugate (ADC) trastuzumab emtansine (T-DM1), which was associated with PFS and OS improvement when compared to capecitabine plus lapatinib in patients with metastatic HER2+ pretreated with trastuzumab and a taxane.^6^

Recently, the therapeutic scenario for patients with HER2+ mBC has been revolutionized with the introduction of a new ADC, namely Trastuzumab deruxtecan (T-DXd), which has been specifically engineered to combine an anti-HER2 monoclonal antibody (MoAb) function with the delivery of a potent topoisomerase inhibitor, namely DXd, to cancer cells.^7^

In the phase II trial DESTINY-Breast01 (DB-01), T-DXd demonstrated unprecedented efficacy in heavily pretreated patients with HER2+ mBC. Median PFS registered was 19.4 months associated with a median response duration of 20 months.^8,9^ Moreover, the phase III trial DESTINY-Breast03 (DB-03) demonstrated that T-DXd is also associated with superior PFS and OS when compared to T-DM1 as a second-line therapy for patients with HER2+ mBC progressing on first-line taxane-trastuzumab-pertuzumab.^10,11^ Based on results of the DB-03 trial, T-DXd will become the standard-or-care second-line therapy for patients with HER2+ mBC. More recently, T-DXd demonstrated a statistically significant and clinically meaningful improvement in PFS and OS versus treatment of physician’s choice in patients with HER2+ unresectable and/or mBC previously treated with T-DM1 (DESTINY-Breast02 trial).^12^

However, despite this impressive clinical progress and the rapidly emerging role of T-DXd in HER2+ BC treatment, real-world data about safety and efficacy of T-DXd in routine clinical practice are lacking. Real-world information are urgently needed to better understand the management of this new treatment and its real activity in unselected patients.

In this analysis, we reported the results of a retrospective multicenter study, the DE-REAL study, which investigated the safety, antitumor activity, and efficacy of T-DXd in an unselected real-world population of patients with HER2+ mBC.

## Material and Methods

### Patient Population and Enrollment Criteria

The medical records of 12 Italian referral hospitals were reviewed to identify patients with HER2+ mBC treated with T-DXD. Eligible patients were aged 18 years or older and were required to have a diagnosis of unresectable/metastatic HER2-positive BC. HER2 positivity was determined locally, and defined as 3+ immunohistochemical (IHC) staining or 2+ IHC staining and positive fluorescence in situ hybridization test (FISH).^13^ Patients who received at least one administration of T-DXd as any therapy line for advanced disease were included in the analysis.

### Planning Schedule

Patients received T-DXd (5.4 mg per kilogram of body weight) every 3 weeks, until progression or unacceptable toxicity.

### Study Objectives and Endpoints

The primary objective of the study was to evaluate the efficacy of T-DXd in a real-world population of patients with HER2-positive mBC. Radiological response was locally evaluated and the tumor assessment was performed every 3 to 4 months according to national and local guidelines.^14^ All patients were evaluated with full body CT scan. Bone scan, brain MRI, and PET scan were included if clinical indicated. In presence of visceral, measurable disease, RECIST 1.1 criteria were applied locally to evaluate the radiological response.^15^ Considering the real-world data collected in our study, we selected the real-world PFS (rwPFS) as primary endpoint.^16^

RwPFS was defined as the time from start of treatment to evidence of disease progression or death, whichever occurred first, in a clinical practice context, with no strict protocols for radiological evaluations and centralized review of images.^17,18^ Patients without an rwPFS event were censored at the last time they were known to be alive and free from progression.

Secondary end-points were overall response rate (ORR—defined as complete plus partial responses), disease control rate (DCR), and overall survival (OS).

The proportion of patients that achieved complete response, partial response, and stable disease defined the DCR.

OS was defined as the time from start of T-DXd treatment to death; patients without an OS event were censored at the last time they were known to be alive. Milestone rwPFS and OS were evaluated at 6, 12, and 18 months, respectively.

Furthermore, adverse events (AEs) and safety profile were evaluated. Treatment-related AEs collected in the referral centers were categorized and graded according to the National Cancer Institute Common Terminology Criteria for Adverse Events (NCI CTCAE), version 5.0.^19^

### Statistical Analysis

Mean ± standard deviation and number (percentages) were used for summarizing data. Continuous variables were analyzed using independent *t*-test, while categorical variables were studied using chi square or Fisher’s exact test. Univariate and multivariate analysis were performed to identify independent factors that had an impact on real-world progression-free survival (rwPFS) and overall survival (OS). The Kaplan-Meier method was used to estimate the distribution of rwPFS and OS. Differences among groups in time-to-event endpoints were tested with unstratified log-rank tests. Hazard ratios (HR) with their relative 95% confidence intervals (95% CI) were estimated with Cox regression analyses.

## Results

### Study Population

Between April 2021 and October 2022, 143 patients were enrolled in the De-REAL study. Median patient age was 66 (range 37 to 90), and 4 patients were male (3%). Patients’ characteristics are reported in Table 1. Across the patient population, 108 (75%) patients had hormone receptor positive (HR+/HER2+) tumors, whereas the remaining 35 (25%) patients had HR-negative/HER2-positive (HR−/HER2+) BC. T-DXd was administered as first or second-line in 20 patients (14%), while 123 patients (86%) received treatment as third or subsequent lines. The median number of previous lines of therapy for metastatic disease was 4 (range 1 to 11). At the time of data cutoff, 78 of 143 patients (54%) were still receiving T-DXd.

**Table 1.** Patients’ characteristics.

Patients’ characteristics	Patients *N* = 143 (%)
Age—years	
Median	66
range	37-90
Sex	
Female	139 (97)
Male	4 (3)
N of comorbidities	
0	101 (70)
1	21 (15)
≥2	21 (15)
Hormone receptor	
Positive	108 (75)
negative	35 (25)
HER2 status	
ICH 3+	111 (78)
ICH 2+, ISH positive	32 (22)
Line of treatment with T-DXd	
First	4 (3)
Second	16 (11)
Third	42 (29)
Subsequent	81 (57)
Average (range)	4 (1-11)
Previous anti HER2 treatments	
Trastuzumab	143 (100)
Pertuzumab	103 (72)
TDM-1	111 (78)
Lapatinib	60 (42)
Neratinib	10 (7)
Tucatinib	3 (2)
Visceral disease	
Yes	84 (59)
No	59 (41)
CNS metastases	
Yes	36 (25)
No	107 (75)

Abbreviations: N: number of patients; HER2: human epithelial receptor 2; ICH: immunohistochemistry; ISH: in situ hybridization.

### Outcomes

**Figure 1.**
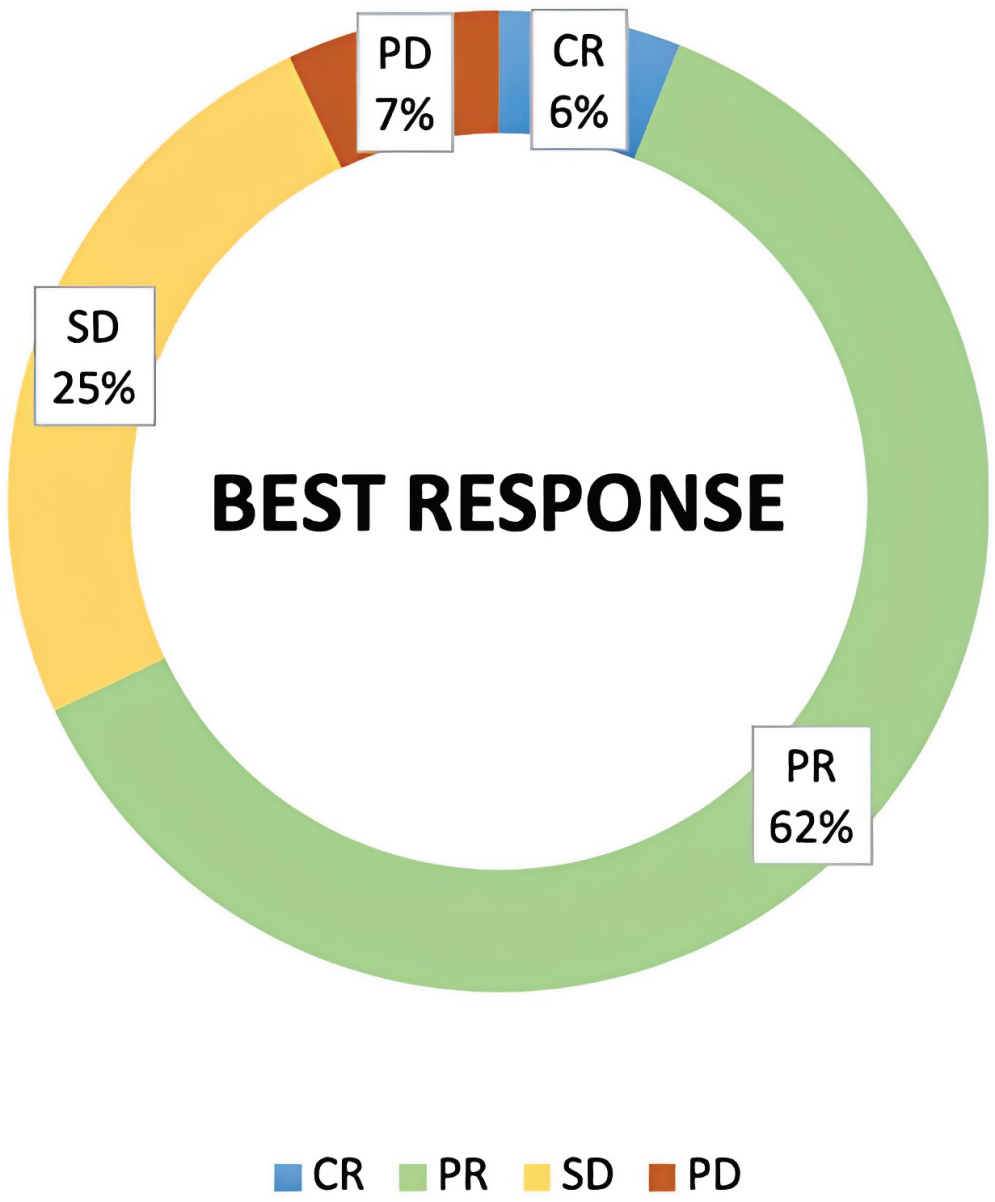
Best response in the overall population. Response distribution according to RECIST 1.1 criteria.

**Figure 2.**
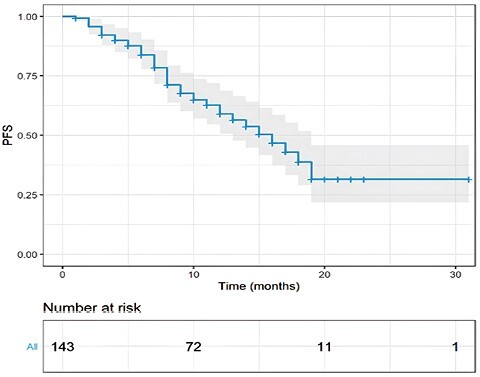
PFS in the whole study population. Kaplan-Meier estimates of PFS in the overall population. mPFS was 16 months. The gray area represents the confidence interval. Tick marks represent data censored at the last time the patient was known to be alive.

**Figure 3.**
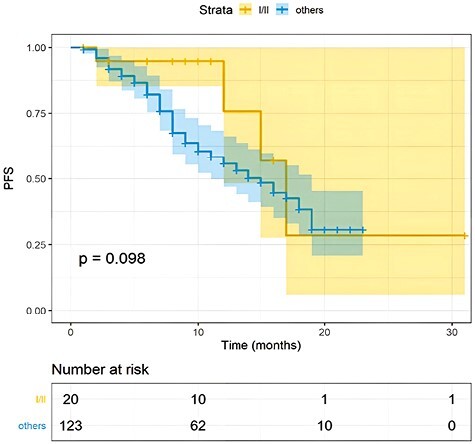
PFS according to T-DXd treatment line. Kaplan-Meier estimates of PFS in the I/II line versus subsequent lines. mPFS was 17 versus 15 months (*P* = .098). The colored area represents the confidence interval. Tick marks represent data censored at the last time the patient was known to be alive.

**Figure 4.**
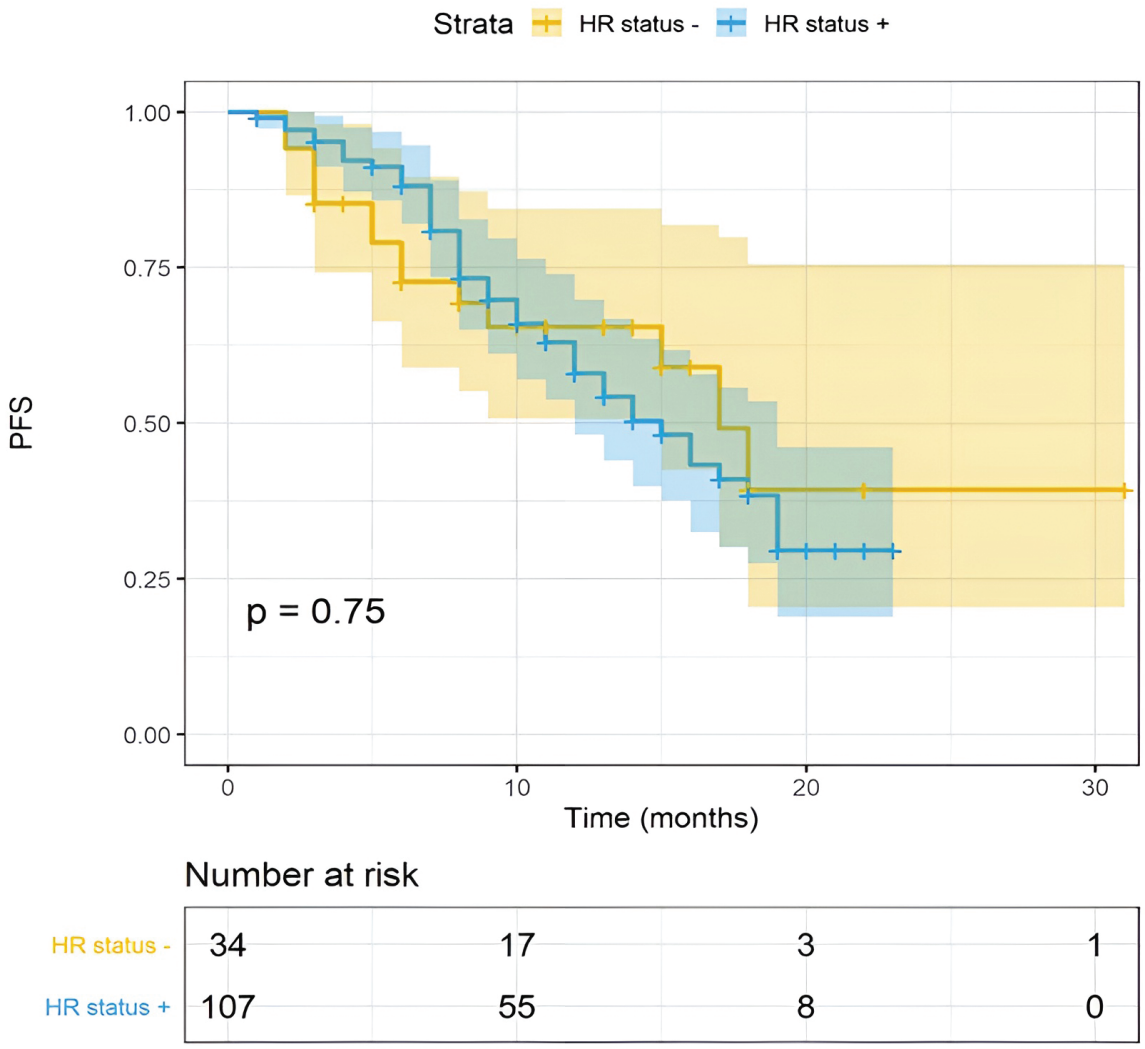
PFS according to HR status. Kaplan-Meier estimates of PFS in HR- versus HR+. mPFS was 17 versus 15 months (*P* = .75). The colored area represents the confidence interval. Tick marks represent data censored at the last time the patient was known to be alive.

One hundred twenty-three patients had measurable disease and, among these, the observed ORR was 68%. Of these patients, 9 (6%) achieved a complete response (CR), while 74 (62%) had a partial response (PR) during T-DXd therapy (Table 2; Fig. 1). Another 25% of patients (*n* = 30) reported a stable disease (SD), while primary resistance to T-DXd was detected in 7% of cases (*n* = 9). The disease control rate (DCR) registered in our population was 93% (6% RC, 62% PR, and 25% SD).With a median follow-up of 12 months (range, 2 to 31), the overall median rwPFS was 16 months (95% CI, 13-19 months; Fig. 2). Regarding milestone rwPFS, 84%, 59%, and 39% of patients were free from progression at 6, 12, and 18 months after treatment initiation, respectively. Patients receiving T-DXd as first/second line of treatment for advanced disease showed a numerical longer rwPFS when compared to patients treated in more advanced lines (17 vs. 15 months, HR = 2.24, 95% CI, 0.89-3.96, *P* = .098; Fig. 3). However, this difference did not reach statistical significance. Of note, T-DXd was associated with similar rwPFS in patients with HR+ or HR− disease (HR: 0.92, 95% CI, 0.51-1.67, *P* = .78; Fig. 4).

**Table 2.** Response outcomes according to RECIST 1.1 criteria.

RECIST response	Patients (%)
Complete Response (CR)	9 (6%)
Partial Response (PR)	74 (62%)
Stable disease (SD)	30 (25%)
Progessive Disease (PD)	10 (7%)

Response Outcomes	%
Overall response rate (ORR)	68
Disease control rate (DCR)	93

**Figure 5.**
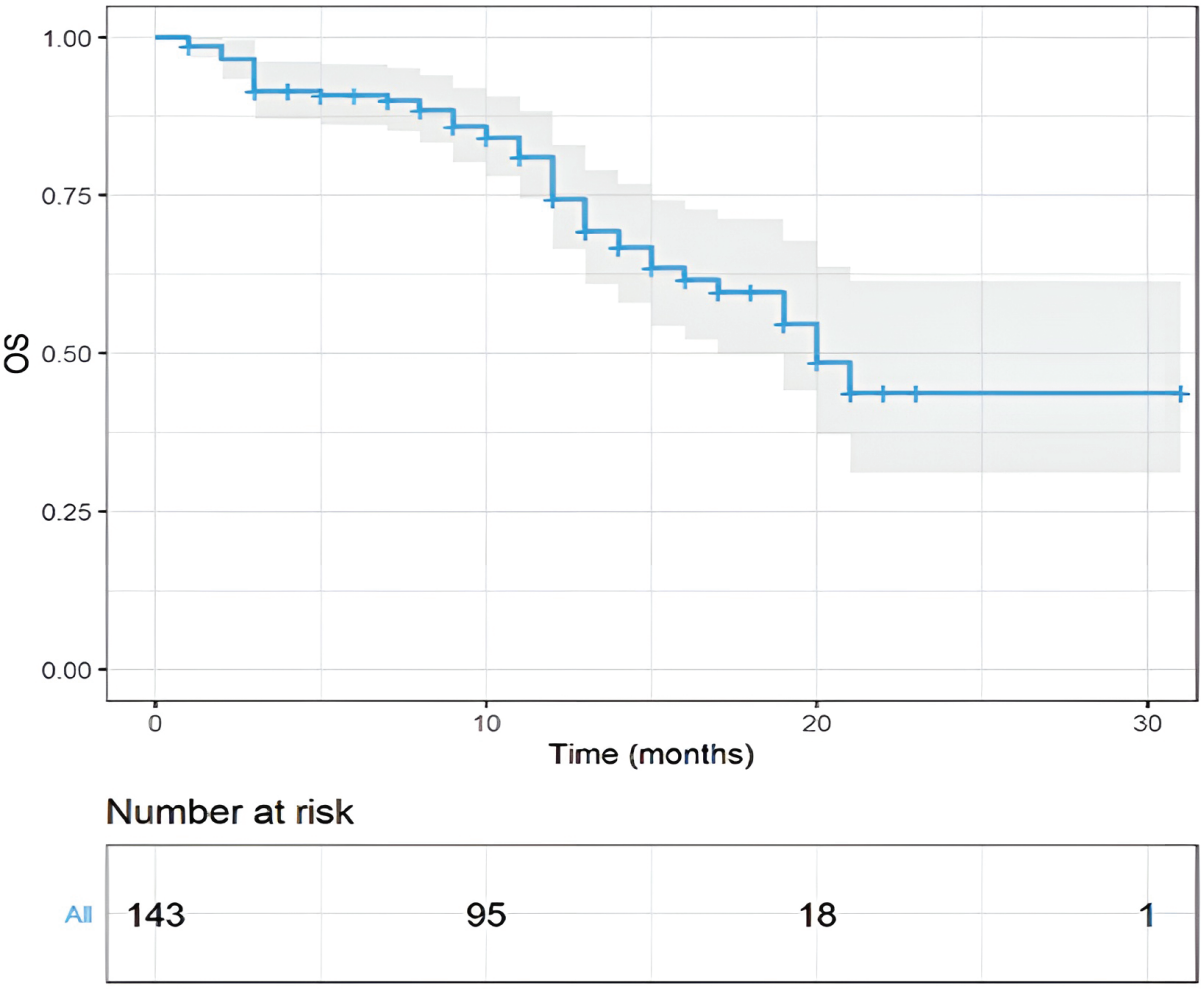
OS in overall population. Kaplan-Meier estimates of OS in the overall population. mOS was 20 months. The gray area represents the confidence interval. Tick marks represent data censored at the last time the patient was known to be alive.

Median overall survival (OS) in our patient population was 20 months (95% CI, 19-31 months; Fig. 5). At 18 months, the OS rate was 59%. Among patients receiving T-DXd as a first/second line of therapy, 12 month OS rate was 89%, while it was 73% in patients treated with T-DXd as subsequent lines of treatment (HR = 1.63, 95% CI, 0.58-4.03, *P* = .39).

### Safety

Table 3 summarizes the incidence of treatment-related AEs reported in the study. Of the 143 patients treated with T-DXd, 59% experienced at least one adverse event (*N* = 84). Overall, 69% of patients (*N* = 58) experienced low-moderate grade toxicities (G1/G2). Nausea was the most common AE (33%, any grade), and it was reported in 47 patients. Neutropenia and fatigue were also frequent, occurring both in 21% of cases (*N* = 30, any grade). All patients reporting fatigue/asthenia had G1/G2 toxicity, while 50% of patients with neutropenia experienced G3/G4 events (*N* = 15). Alopecia was registered only in 9 patients, and no severe grade was found. Anemia was an infrequent AE, occurring in 9 cases (6%), with only one G3 event detected. Liver toxicity and diarrhea were also uncommon in our population (5% and 1%, respectively). Severe AEs (sAEs) occurred in 18% of all patients receiving T-DXd (*N* = 26/143). The most frequent grade 3 or 4 toxicity was neutropenia (17%). Interstitial lung disease (ILD) was a rare AE, and it was reported in only 3/143 patients (2%). Nausea/vomiting were severe in only 2% of cases. AEs led to T-DXd discontinuation in 3% of patients and to a dose reduction in 26% of patients (*n* = 37). No drug-related fatal toxicity was reported in our analysis. Of note, the rwPFS and OS rates were not affected by dose reduction (ie, HR = 1.13, 95% CI, 0.65-1.96, *P* = .66 and HR = 1.59, 95% CI, 0.82-2.97, *P* = .18, respectively) or any-grade toxicity during T-DXd (HR = 1.59, 95% CI, 0.82 to 2.97, *P* = .18). Similarly, no difference was detected in survival outcomes according to toxicity grade (G1/G2 vs. G3/G4, HR = 0.89, 95% CI, 0.42 to 1.85, *P* = .74).

**Table 3.** Most common drug-related adverse events in the overall population.

Event	Any grade *N* (%)	Grade 3 or 4 *N* (%)
Nausea	47 (32%)	2 (1.3%)
Fatigue	30 (20%)	0 (0%)
Neutropenia	30 (20%)	15 (10%)
Decrease platelet count	12 (8%)	0 (0%)
Anemia	9 (6%)	1 (0.6%)
Alopecia	9 (6%)	0 (0%)
Increased liver enzymes	8 (5%)	0 (0%)
ILD	3 (2%)	3 (2%)
Diarrhea	2 (1%)	1 (0.5%)

Abbreviations: N: number of patients; ILD: interstitial lung disease.

The ORR was comparable in patients with and without any-grade toxicity (67% vs. 68%, respectively, *P* = .74) and with low- and high-grade toxicities (68% vs. 66%, respectively, *P* = .63). No difference in ORR was reported in patients receiving T-DXd as I/II versus subsequent lines (72% vs. 67%, *P* = .88).

## Discussion

We reported the results of the multicenter, retrospective Italian study DE-REAL, which investigated the safety and efficacy of T-DXd in a real-world population of patients with HER2+ mBC. We showed that T-DXd is safe, active and effective in patients with HER2+ mBC. Of note, T-DXd was similarly effective when used in different lines of therapy, and regardless of HR status. Overall, our findings support the efficacy of T-DXd in a real world setting, thus providing additional evidence of clinical relevance of this novel drug for patients with HER2+ mBC.

T-DXd showed unparalleled efficacy in metastatic HER2+ BC previously exposed to other standard anti-HER2 therapies.^8-12^ Although T-DXd has massively entered our daily practice based on excellent results of prospective clinical trials, the safety, feasibility, and antitumor efficacy of T-DXd in a real-world population of patients with HER2+ mBC remains unknown.

The DE-REAL study tried to fill this knowledge gap by collecting information about the real-world safety and efficacy of T-DXd in a real world population of patients with HER2+ mBC that does not fulfill the strict criteria that are commonly used in DESTINY-Breast clinical trials.^8-12^

Notably, safety and efficacy results of T-DXd in our real-world cohort of patients treated with T-DXd are comparable to the results of clinical trials. In more detail, the single-arm DB-01 and the randomized phase III DB-02 clinical trials showed median PFS of 19.4 months and 17.8 months and ORR of 61.4% and 70%, respectively, in heavily pretreated patients with HER2+ mBC receiving T-DXd.^8,9,12^ Moreover, a significant improvement in PFS was shown with T-DXd versus TDM-1 for patients with HER2-positive mBC pretreated with trastuzumab and taxane in DB-03 trial (28.8 vs. 6.9 months, 95% CI, 22.4-37.9).^10,11^ Patients enrolled in DB-01 had received a median number of 6 systemic treatments for advanced disease before being treated with T-DXd.^8,9^ Regarding DB-02 trial, patients had been exposed to a median number of 2 lines of systemic therapy before receiving T-DXd, whereas in DB-03 trial the 65% of patients received T-DXd as first/second line.^10-12^ In our study, the median number of prior lines was 4, and median rwPFS was 16 months, which is in line with results of DB-01 and DB-02 trials, where T-DXd was administered after a different number of prior systemic lines of therapy. In the DE-REAL study, only a small fraction of population (20 patients, 14%) received T-DXd in a first/second line setting and could be compared with the population of the DB-03 trial.^10,11^ Of note, among patients receiving T-DXd as early treatment, only one has progressed before the 12th month. Fifteen patients had a follow-up longer than 12 months with no progression, and 4 had a follow up shorter than 12 months. Collectively, in this subgroup, the 12-month rwPFS rate was 75%, 12-months OS rate 89% and ORR 70%. Although the small percentage of patients receiving T-DXd in early settings in our cohort, these findings were comparable to DB-03 results (12-months PFS of 75.2%, 12-months OS of 94.1%, and ORR 79% reported, respectively).^10,11^

These results, together with our data showing that T-DXd is similarly effective in patients treated in first/second versus more advanced treatment lines, suggests that HER2+ mBC sensitivity to T-DXd is not significantly affected by the number of prior lines of anti-HER2 therapies, but it could reflect intrinsic tumor sensitivity or resistance to this agent. This, in turn, could be a function of the capability of tumor cells to bind T-DXd (based on HER2 expression on tumor cell plasma membranes) and/or the intrinsic sensitivity of tumor cells to the payload DXd.

In our study, the observed ORR of 68% was also promising, and in line with results of known findings. These results confirm consistent antitumor activity of T-DXd also in later lines of treatment and in patients with comorbidities and high disease burden. As expected according to literature, no difference was shown in outcomes regarding to HR status.^8,10^

Median OS in DB-01 and in DB-02 was 24.6 and 39.2 months, respectively, while the median OS in our cohort was 20 months.^8,9,12^ However, OS data in our study are still immature, and a longer follow-up is needed to draw more definitive conclusions about the impact of T-DXd on OS in a real-world population of patients with HER2+ mBC.

Differences in the study population that might have influenced the results needs to be critically discussed. First, patients enrolled in DB-01 had a higher median number of previous treatment regimens when compared to patients included in our real-world cohort; on the other hand, patients enrolled in DB-02 were less heavily pretreated. In addition, patients enrolled in our real-world cohort had older age than patients enrolled in both clinical trials (66y vs. 55y in DB-01 and 54y in DB-02, respectively).^8,9,12^ These differences may explain the similar PFS outcomes, but potentially lower OS in the DE-REAL cohort as compared patient cohorts included in DESTINY-Breast trials.

Another relevant finding of our real-world is that the reported rate of toxicities and severe toxicities was lower than in published trials.^9-12,20^ Indeed, despite the fact that we enrolled patients with older median age, we reported lower bone marrow toxicity (ie, anemia, decreased neutrophil count) when compared to the observed rates of bone marrow toxicities in published clinical trials. This finding may depend on several factors: (1) retrospective analyses, such as the DE-REAL study, are limited by an under-reporting of the incidence and grading of AEs, which reflects the retrospective collection of safety data; (2) the increasing experience in the prevention or management of T-DXd-related toxicities in the clinical practice may have reduced the incidence of some severe AEs that are typical of T-DXd, such as nausea and ILD; (3) in real-world analyses, dose reductions are more frequent, mostly due to less stringent protocols; this, in turn, may result in lowered toxicities, including hematological and non-hematological ones. Lower non-hematologic toxicity (ie, diarrhea, nausea, fatigue) was also observed in our cohort with low rates of grade ≥3 events. In particular, we reported a lower incidence of nausea/vomiting if compared to registration trials. However, those results may be influenced by an increased and better management of antiemetic prophylaxis among centers adopted in clinical practice, probably in response to high nausea/vomiting reported in DESTINY-Breast trials.^8-12^ Furthermore, subjective toxicity assessment in real-world cohorts may be affected by underreporting in medical records, as already shown in literature.^21,22^

Both treatment-related adverse events (AEs) and dose reduction were studied in relation to their impact on the patients’ survival outcomes and treatment response. Our retrospective analysis showed that neither treatment-related toxicities nor dose reduction appeared to significantly affect patients’ rwPFS and OS. Moreover, ORR was similar in patients that experienced toxicities of any grade compared to population without AEs.

Of note, the occurrence of AEs during T-DXd or the decision to reduce the treatment dosage did not seem to have a substantial negative impact on the patients’ chances of survival in clinical practice, at least based on the data analyzed.

Similar real-world experiences have been conducted in other countries. In the TREX-Old retrospective registry, colleagues evaluated the toxicity of T-DXd in elderly patients (≥70 years). Overall, any-grade treatment-related AEs occurred in 19 patients (70%). Among them, nausea was the most common AE (37%), followed by asthenia (18%) and 2 out of 14 patients developed grade 3 or 4 AEs (14%). Eight patients (30%) started with a dose-reduction, and 8 patients (30%) had secondary dose adjustment.^23^ The results were similar to those in our study with 37% nausea and 18% asthenia, as common toxicities. Another single-center experience reports 13% severe AEs.^24^ A UK real-world data collection reported an higher number of discontinuation but a similar efficacy compared to DB-02.^25^ Clinically relevant efficacy was confirmed in all these unselected, real-world populations. A strength of our study consist in the multicenter effort to collect data about the activity and the safety of T-DXd in a heavily pretreated, real-world cohort of patients with HER2+ mBC who are also older than patients typically included in randomized clinical trials. Moreover, the population of our study is greater compared to other real-world experiences, increasing the reliability of our findings. The following are potential study limitations: rwPFS data may have been over-estimated as a result of longer time intervals between subsequent radiological assessments in real-world assessments as compared to clinical trials, in which tumor re-evaluation typically occurs every 6-8 weeks, at least during the first 1-2 years of treatment.^22^ In addition, other limitations of DE-REAL study consist in limited sample size, the lack of centralization of radiological evaluations, the different timing of imaging assessment among centers and the possibility of an underestimation of AEs.

## Conclusions

In conclusion, we confirm the excellent efficacy and safety outcomes of T-DXd in a real-world population of Italian patients with heavily pretreated HER2-positive mBC. Real-world multicenter studies including higher numbers of patients and with longer follow-up are needed to confirm findings of our study, as well as to identify patient- and tumor-related variables associated with the efficacy of T-DXd in patients with HER2+ mBC.

## Data Availability

The dataset analyzed during the current study are available from the corresponding author on reasonable request (simone.scagnoli@uniroma1.it).
